# The multicentre south European study 'Helios'. I: Skin characteristics and sunburns in basal cell and squamous cell carcinomas of the skin.

**DOI:** 10.1038/bjc.1996.274

**Published:** 1996-06

**Authors:** R. Zanetti, S. Rosso, C. Martinez, C. Navarro, S. Schraub, H. Sancho-Garnier, S. Franceschi, L. Gafà, E. Perea, M. J. Tormo, R. Laurent, C. Schrameck, M. Cristofolini, R. Tumino, J. Wechsler

**Affiliations:** Registro de Càncer de Granada, Escuela Andaluza de Salud Publica, Spain.

## Abstract

The aim of this study was to investigate constitutional and environmental determinants of non-melanocytic skin cancer among different populations from south Europe. Between 1989 and 1993 we interviewed incident cases and a random population sample of controls from five centres where a cancer registry was operating, whereas we selected a sample of hospital-based cases and controls from three other centres. Controls were stratified according to the age and sex distribution of cases. In all, 1549 cases of basal cell carcinoma (BCC), 228 of squamous cell carcinoma (SCC) and 1795 controls were interviewed. Both cancers affected primarily sun-exposed sites such as face, head and neck, but the prevalence of BCC on the trunk was higher than for SCC. Pigmentary traits such as hair and eye colour as well as tendency to sunburn were strong and independent indicators of risk for both BCC and SCC. In SCC, adjusted odds ratios (ORs) ranged from 1.6 for fair hair colour to 12.5 for red hair. Light-blonde hair entailed a risk of about 2 for BCC. Pale eye colour was associated with a risk of 1.8 for SCC and 1.4 for BCC. Subjects who always burn and never tan showed an adjusted OR of 2.7 for BCC and 2.0 for SCC. A history of sunburns and a young age at first sunburn were associated with an increased risk for BCC only (OR 1.7). Pigmentary traits and sun sensitivity of the skin confirmed their role as risk indicators. The effect of sunburns, as an indicator of both exposure and sun sensitivity of the skin, is less clear. Nevertheless, its association with BCC suggests, by analogy with melanoma, a relationship with intense sun exposure. Conversely, SCC would require prolonged exposure to sunlight.


British Journal of Cancer (1996) 73, 1440–1446
© 1996 Stockton Press All rights reserved 0007–0920/96 $12.00

### The multicentre south European study '*Helios*' I: skin characteristics and sunburns in basal cell and squamous cell carcinomas of the skin

R Zanetti[1], S Rosso[1], C Martinez[2], C Navarro[3], S Schraub[4], H Sancho-Garnier[5], S Franceschi[6], L Gafà[7], E Perea[2], MJ Tormo[3], R Laurent[8], C Schrameck[9], M Cristofolini[10], R Tumino[7] and J Wechsler[11]

*[1]Registro Tumori Piemonte, via S. Francesco da Paola 31, 10123 Turin, Italy; [2]Registro de Càncer de Granada, Escuela Andaluza de Salud Publica, Campus de Cartuja, 18080 Granada, Spain; [3]Registro de Càncer de Murcia, Consejeria de Sanidad y Asuntos Sociales, Ronda de Levante 11, 30008 Murcia, Spain; [4]Registre des Tumeurs du Doubs, C.H.R. Jean Minjoz, Boulevard Fleming, 25030 Besançon, France; [5]Département de l'Information Médicale, Hôpital Gaston Doumergue, 5 Rue Hoche, 30006 Nîmes, France; [6]Centro di Riferimento Oncologico, via Pedemontana Occidentale 12, 33081 Aviano, Italy; [7]Registro Tumori di Ragusa, Piazza Igea 2, 97100 Ragusa, Italy; [8]Service de Dermatologie, Hôpital Saint Jacques, 25030 Besançon, France; [9]Service de Biostatistique, Institut Gustave Roussy, Rue Camille Desmoulins, 94805 Villejuif, France; [10]Dipartimento di Dermatologia, Ospedale S. Chiara, Largo Medaglie d'Oro, 38100 Trento, Italy; [11]Service d'Anatomie et de Cytologie Pathologiques, Hôpital Henri Mondor, 94010 Créteil, France.*

**Summary** The aim of this study was to investigate constitutional and environmental determinants of non-melanocytic skin cancer among different populations from south Europe. Between 1989 and 1993 we interviewed incident cases and a random population sample of controls from five centres where a cancer registry was operating, whereas we selected a sample of hospital-based cases and controls from three other centres. Controls were stratified according to the age and sex distribution of cases. In all, 1549 cases of basal cell carcinoma (BCC), 228 of squamous cell carcinoma (SCC) and 1795 controls were interviewed. Both cancers affected primarily sun-exposed sites such as face, head and neck, but the prevalence of BCC on the trunk was higher than for SCC. Pigmentary traits such as hair and eye colour as well as tendency to sunburn were strong and independent indicators of risk for both BCC and SCC. In SCC, adjusted odds ratios (ORs) ranged from 1.6 for fair hair colour to 12.5 for red hair. Light-blonde hair entailed a risk of about 2 for BCC. Pale eye colour was associated with a risk of 1.8 for SCC and 1.4 for BCC. Subjects who always burn and never tan showed an adjusted OR of 2.7 for BCC and 2.0 for SCC. A history of sunburns and a young age at first sunburn were associated with an increased risk for BCC only (OR 1.7). Pigmentary traits and sun sensitivity of the skin confirmed their role as risk indicators. The effect of sunburns, as an indicator of both exposure and sun sensitivity of the skin, is less clear. Nevertheless, its association with BCC suggests, by analogy with melanoma, a relationship with intense sun exposure. Converseley, SCC would require prolonged exposure to sunlight.

**Keywords:** basal cell carcinoma; squamous cell carcinoma; pigmentation; sun burns; skin cancer

Non-melanocytic skin cancer is one of the commonest tumours in white populations. Standardised incidence rates in men, as measured by cancer registries in the late 1980s, ranged from about 40 cases per 100 000 in various European countries, to 100 and 200 cases per 100 000 respectively in North America and Australia (Parkin *et al.*, 1992). It is generally believed that incidence rates, as reported by cancer registries, are underestimated, because current notification systems miss some cases diagnosed and treated only as outpatients. Two surveys carried out in Australia, the highest risk area worldwide, found annual incidence rates to be over 1000 cases for 100 000 inhabitants (Kricker *et al.*, 1990; Stenbeck *et al.*, 1990).

Non-melanocytic skin cancers are classified into two major groups: basal cell carcinoma (BCC) and squamous cell carcinoma (SCC). BCC is the most common cancer type in white populations, occurring rarely among black Africans, among whom SCC is a little more common that BCC though still rare (Oettlé, 1963). As reported by some surveys, the incidence of BCC has increased in British Columbia (Gallagher *et al.*, 1990), USA (Fears and Scotto, 1982), the Netherlands (Coebergh *et al.*, 1991) and Switzerland (Levi *et al.*, 1988) during the last two decades. In addition, these upward trends were especially marked for BCC on the trunk, while for SCC, they were limited to the head, face and neck (Gallagher *et al.*, 1990; Fears and Scotto, 1982; Coebergh *et al.*, 1991).

Risk factors for BCC and SCC have been examined in a few analytical studies. Pigmentary characteristics, skin sensitivity to sun and sun exposure emerged as the major risk factors, although the relationship among these highly correlated indicators is still controversial (Kricker *et al.*, 1994). None of the studies on skin cancer recently reviewed had sufficient SCC cases to test for differences between BCC and SCC with an adequate statistical power (IARC, 1992). Subsequently, a few other analytical studies have been published (Ron *et al.*, 1991; Kubasiewicz *et al.*, 1991), and recent analyses have included data on the qualitative and quantitative relationship between risk of BCC (Kricker *et al.*, 1995*a*, *b*; Gallagher *et al.*, 1995*a*), of SCC (Gallagher *et al.*, 1995*b*) and sun exposure.

To elucidate further the aetiology of non-melanocytic skin cancers, in 1989 we planned a case–control study of sufficient size to evaluate the causal role of sun exposure, constitutional factors, occupational and iatrogenic exposures on SCC and BCC separately. The study was implemented among south European populations in order to increase variability of phenotypes and lifestyles and provide insight into the epidemiology of skin cancer in a population still little studied. In this paper we present results on pigmentary traits, skin sensitivity to sun exposure and sunburns.

Correspondence: R Zanetti, Registro Tumori Piemonte, Unità di Epidemiologia dei Tumori, via San Francesco da Paola 31, 10123 Turin, Italy
Received 21 August 1995; revised 20 December 1995; accepted 8 January 1996

### Methods and subjects

### *Cases ascertainment*

The recruitment of cases and controls took place in seven south European regions between November 1989 and June 1993: Turin (north-west Italy), Trento (north-east Italy), Ragusa (Sicily), Villejuif and Créteil (Paris), Besançon (Franche-Comté, France), Murcia (south-east Spain) and Granada (Andalusia, Spain). Population-based cancer registries are operating in Turin, Ragusa, Besançon, Murcia and Granada, covering a total population of over 3.5 million inhabitants. In these areas, all incident cases between 20 and 70 years of age with a diagnosis of BCC, SCC and carcinoma of skin adnexa, as identified by cancer registries' notification systems, were considered eligible. In Trento, cases were identified at the Dermatology Service of the main regional hospital, where virtually all skin cancer cases of the area are diagnosed and treated. In Paris, case recruitment was carried out in two specialised centres: Institut Gustave Roussy, Villejuif and Hôpital Henri Mondor, Créteil. Dermatologists or family physicians asked cases to consent to an interview about 'lifestyle and health'. When contact was made directly, informed consent was asked before interviews. In population-based centres cases were interviewed at dermatological clinics or at home, while in hospital-based centres cases were interviewed during their stay in hospital.

We collected the histological report of all interviewed cases, together with the slides whenever possible. A panel consisting of one pathologist from each centre was constituted in order to validate and evaluate reproducibility of morphological diagnoses. Each panel's participants reviewed diagnoses blindly, exchanging slides with each other and discussing discordant cases in plenary sessions. Cases with more than one concurrent skin cancer were assigned to the first occurring or diagnosed cancer.

### *Controls sampling*

We drew the control group as an age- and sex-stratified random sample of respective general populations in areas covered by cancer registries, with strata proportional to the age and sex distribution of the skin cancers; samples were drawn from electoral rolls in Ragusa and Besançon, and from the population registries in Turin, Murcia and Granada. Control sampling was hospital-based in Paris and Trento, excluding patients with cancer or skin diseases. We contacted population controls by mail and interviewed them at home, at work or at cancer registry locations, whereas hospital controls were approached and interviewed during their stay in hospital.

### *Assessment of exposure*

All subjects who agreed to collaborate were interviewed by a trained interviewer with a standard questionnaire. All interviewers were trained by the same senior interviewer, who replicated the same 4-day course in Italy, France and Spain, paying particular attention to ascertain exposures in a similar way among cases and controls in order to minimise misclassification. The senior interviewer checked their performances in the first interviews and then systematically reviewed occupational histories and internal consistency of outdoor activity histories. After data gathering in the central data base, quality checking was performed on missing values, extreme values, etc.

The questionnaire covered host factors (skin characteristics, pigmentary traits), history of past and present places of residence, life-long exposure to sunlight, occupational history, dermatological history, cosmetic habits (sunscreens, sunlamps, sand and mud bath), use of immune-suppressor and radiological exposures.

Measures of pigmentary traits were taken by assessing hair and eye colours. We graded hair colour against 11 samples of human hair provided by a cosmetic firm (L'Oréal). For subjects who were bald, had gone grey or dyed their hair, we assessed eyebrow hair, which, we consider, keeps its natural colour longer than scalp hair, or, if not possible, asked subjects to select the hair colour that would have matched their original or natural colour best. Eye colour was assessed on a three-level scale (black and brown; green; blue and grey).

We did not measure skin colour directly because the two methods we tested (sample photos and direct reflectance measurement with opto-electronic colorimeter) turned out to be both unreliable and difficult to implement. Indeed, we considered grading skin colour against a set of six sample photos, and a direct reflectance measurement (Chroma-meter, Minolta). During the pilot period, the first method proved to be highly unreliable because of inter-observer variance. The second method was tested on a sample of about 50 subjects and proved to be highly variable according to skin site, age and sex of subjects. This is not surprising and is consistent with previous findings with objective measurements. The skin colour is not only dependent on pure melanin pigmentation, but is also influenced by body hair, thickness, moisture, superficial diffusion of blood vessels and tanning. Moreover, objective measurements are only weakly associated with skin cancers and actinic lesions, suggesting that it is not skin colour, but rather a combination of pigmentation and sensitivity to sun, that induces skin tumours (Green and Martin, 1990; Kricker *et al*., 1991).

Skin characteristics were measured by asking questions about reaction to sun exposure and sunburns in childhood. Reaction to sun exposure was measured on a four-level scale and ranged from subjects who always tan and never burn to subjects who always burn when exposed to sun. As reaction to sun exposure varies during life according to the degree of melanin protection and skin thickness, we asked patients to report their skin reaction experience at 20 years old. Past experience of sunburns was assessed by asking questions about number of sunburns and age at first sunburning.

### *Scales construction and data analysis*

In this analysis we evaluated the effect of skin characteristics and sunburns during different outdoor activities on both BCC and SCC. Since analysis was conducted on BCC and SCC separately and given the different control sampling schemes, we controlled the residual confounding effect of design variables by means of odds ratios adjusted for age, sex, and centre. Each factor was then analysed, including significant pigmentary traits and skin characteristics in unconditional logistic models with design variables to establish which factors were independent from all others.

Hair colour, eye colour and skin reaction to sun exposure were all measured on ordinal scales. Point estimations were computed at each level of the scale, then collapsing adjacent classes with similar estimates, in order to increase the efficiency of models, while saving degrees of freedom. Number of sunburns and age at first sunburn were both determined on an interval scale, but given the skewness of their distributions, we applied quartiles of distributions in exposed controls. Further, we tested linear trends of such reduced scales.

The effect was tested of different control sampling bases on odds ratios (population based in five centres and hospital based in three centres); results indicated that parameter estimates tended to aggregate according to national clusters rather than to their sampling basis. For this reason, when it was necessary to deal with more parsimonious models and controlling by centre effect, we grouped centres in three national groups.

The independent effect of risk factors was tested including all variables in a model and then evaluating if there were significant interaction terms. Only significant variables or confounders (i.e. with a relevant effect on other coefficients) were retained. Finally the model was further checked with the appropriate logistic regression diagnostics (Pregibon, 1981).

### Results

We interviewed 1832 cases and 1795 controls. The response rate was 85.8% among cases and 69.3% among controls in population-based centres (Table I). About 8.8% cases refused to be interviewed and 3.0% cases could not be traced because of a change in residence or death. Among controls, 18.5% refused to be interviewed and 7.9% could not be traced. Response in population controls in the collaborating centres ranged from 55.6% in Besançon to 82.9% in Ragusa. A small proportion of cases and controls (about 3%) in the hospital-based centres refused to participate.

Case review by the pathologists' panel resulted in identification of 1549 BCC, 228 SCC and 20 carcinomas of the adnexa (not analysed here). Thirty-five (1.9%) cases already interviewed were then discarded from analysis since case review excluded malignancy. Another 38 cases (2.1%) were misclassified by histological type at the first reading. Further details on diagnostic concordance will be presented in a separate article.

The head was the most common site for both SCC (76.8% in men, 58.8% in women) and BCC (78.1% in men, 76.9% in women) (Table II). The rest of the SCC lesions were markedly different from those of BCC: the second most common site for SCC was lower limbs in women (25.5%) and upper limbs in men (7.9%), whereas for BCC the second most common site of lesions was the trunk in both men (14.1%) and women (10.1%).

*Pigmentary traits and skin characteristics*

Pigmentary traits showed a clear and independent association with both BCC and SCC (Table III), but hair colour had a stronger association than eye colour. In general, SCC exhibited elevated risks for subjects with blonde or red hair. Both BCC and SCC showed a 2-fold increase of risk in people who never tan and always get burned when exposed to sun (Table IV). The increase was not linear with the two intermediate categories showing similar estimates in BCC.

Although people with sun-sensitive skin type tend to avoid sun exposure, they too could have experienced some sunburns. As a consequence, we analysed number of life-long sunburns and age at first occurrence as a mixed indicator of both skin reaction and sun exposure.

Number of sunburns showed a highly significant association with BCC, which was however, attenuated by allowance for pigmentary traits and skin type (Table V). Subjects who experienced sunburns before age 15 had a significant, although moderate, increase of risk for BCC, also after allowance for pigmentary traits and skin type (Table V). In addition, age at first sunburn emerged as a significant risk factor for BCC, even after controlling for number of sunburns, while the effect of number of sunburns was no longer significant when age at first sunburn was included in models. Conversely, number of sunburns and age at first sunburn did not seem to be risk indicators for SCC.

Subjects with fair hair and tendency to burn showed a 5-

**Table I** Recruitment of cases and controls by collaborating centre

| | *Identified* | *Refused interview (%)* | | *Untraceable (including death) (%)* | | *Interviewed (%)* | |
|---|---|---|---|---|---|---|---|
| Cases | | | | | | | |
| Turin | 555 | 93 | 16.8 | 3 | 0.5 | 459 | 82.7 |
| Ragusa | 146 | 15 | 7.5 | 0 | 0.0 | 131 | 92.5 |
| Trento | 149 | – | – | – | – | 149 | 100.0 |
| Granada | 358 | 4 | 1.1 | 11 | 3.1 | 343 | 95.8 |
| Murcia | 374 | 38 | 10.2 | 36 | 9.6 | 300 | 80.2 |
| Besançon | 295 | 33 | 11.2 | 12 | 4.1 | 250 | 84.7 |
| Villejuif | 100 | – | – | – | – | 100 | 100.0 |
| Créteil | 100 | – | – | – | – | 100 | 100.0 |
| Total | 2077 | 183 | 8.8 | 62 | 3.0 | 1832 | 88.2 |
| Controls | | | | | | | |
| Turin | 663 | 232 | 35.0 | 9 | 1.4 | 422 | 63.6 |
| Ragusa | 158 | 19 | 12.0 | 8 | 5.1 | 131 | 82.9 |
| Trento | 141 | – | – | – | – | 141 | 100.0 |
| Granada | 428 | 41 | 9.6 | 38 | 8.9 | 349 | 81.5 |
| Murcia | 397 | 36 | 9.1 | 60 | 15.1 | 301 | 75.8 |
| Besançon | 452 | 124 | 27.4 | 77 | 17.0 | 251 | 55.6 |
| Villejuif | 100 | – | – | – | – | 100 | 100.0 |
| Créteil | 100 | – | – | – | – | 100 | 100.0 |
| Total | 2439 | 452 | 18.5 | 192 | 7.9 | 1795 | 73.6 |

**Table II** Site distribution of basal cell carcinomas and squamous cell carcinomas

| | *Men* | | *Women* | |
|---|---|---|---|---|
| *Site* | *BCC (%)* | *SCC (%)* | *BCC (%)* | *SCC (%)* |
| Head | 668 ( 78.1) | 136 ( 76.8) | 516 ( 76.9) | 30 ( 58.8) |
| Neck | 26 ( 3.0) | 6 ( 3.4) | 20 ( 3.0) | – |
| Trunk | 124 ( 14.1) | 4 ( 2.3) | 68 ( 10.1) | 4 ( 7.8) |
| Abdomen | 13 ( 1.5) | 1 ( 0.6) | 24 ( 3.6) | 1 ( 2.0) |
| Lower abdomen | – | 7 ( 3.9) | 5 ( 0.7) | – |
| Upper limbs | 15 ( 1.7) | 14 ( 7.9) | 10 ( 1.5) | 3 ( 5.9) |
| Lower limbs | 14 ( 1.6) | 9 ( 5.9) | 28 ( 4.2) | 13 ( 25.5) |
| Total | 878 (100.0) | 177 (100.0) | 671 (100.0) | 51 (100.0) |

**Table III** Odds ratios (ORs) of BCC and SCC by pigmentary traits

| | *No. of controls* | *No. of BCCs* | *No. of SCCs* | *BCC OR[a] (95% CI)* | *BCC OR[b] (95% CI)* | *SCC OR[a] (95% CI)* | *SCC OR[b] (95% CI)* |
|---|---|---|---|---|---|---|---|
| Hair colour | | | | | | | |
| Black | 154 | 99 | 12 | 1 (Reference) | 1 (Reference) | 1 (Reference) | 1 (Reference) |
| Brown | 699 | 544 | 80 | 1.24 (0.94–1.64) | 1.16 (0.87–1.53) | 1.59 (0.84–3.02) | 1.51 (0.79–2.88) |
| Light brown | 597 | 514 | 67 | 1.41 (1.06–1.87) | 1.20 (0.89–1.60) | 1.83 (0.95–3.52) | 1.54 (0.80–2.99) |
| Blonde | 253 | 257 | 30 | 1.69 (1.23–3.30) | 1.29 (0.93–1.78) | 2.19 (1.07–4.48) | 1.63 (0.79–3.40) |
| Light blonde | 81 | 121 | 29 | 2.47 (1.68–3.63) | 1.78 (1.20–2.66) | 6.93 (3.25–14.84) | 4.88 (2.24–10.63) |
| Red | 11 | 16 | 10 | 2.37 (1.05–5.35) | 1.58 (0.69–3.62) | 18.02 (6.12–52.98) | 12.50 (4.13–37.86) |
| *P*-value (linear trend) | | | | <0.001 | 0.008 | <0.001 | <0.001 |
| Eye colour | | | | | | | |
| Black/dark brown | 542 | 332 | 39 | 1 (Reference) | 1 (Reference) | 1 (Reference) | 1 (Reference) |
| Light brown/green | 816 | 764 | 106 | 1.52 (1.28–1.81) | 1.37 (1.15–1.64) | 1.80 (1.18–2.76) | 1.75 (1.12–2.71) |
| Blue/hazel/grey | 437 | 453 | 83 | 1.71 (1.41–2.08) | 1.35 (1.09–1.67) | 2.60 (1.66–4.07) | 1.83 (1.11–3.03) |
| *P*-value (linear trend) | | | | <0.001 | 0.002 | <0.001 | 0.049 |

[a] Logistic regression estimates with terms for age, sex and centre. [b] Logistic regression estimates with terms for sex, age, centre, hair colour or eye colour, and skin reaction to sun exposure.

**Table IV** Odds ratios (ORs) of BCC and SCC by sun sensitivity

| *Skin reaction to sun exposure* | *No. of controls* | *No. of BCCs* | *No. of SCCs* | *BCC OR[a] (95% CI)* | *BCC OR[b] (95% CI)* | *SCC OR[a] (95% CI)* | *SCC OR[b] (95% CI)* |
|---|---|---|---|---|---|---|---|
| Tan, no burn | 518 | 299 | 44 | 1 (Reference) | 1 (Reference) | 1 (Reference) | 1 (Reference) |
| Rare burn then tan | 288 | 278 | 27 | 1.72 (1.38–2.13) | 1.69 (1.35–2.10) | 0.97 (0.55–1.71) | 0.91 (0.51–1.62) |
| Often burn then tan | 800 | 665 | 114 | 1.52 (1.26–1.82) | 1.43 (1.19–1.72) | 1.73 (1.14–2.57) | 1.50 (0.98–2.22) |
| Burn, never tan | 184 | 299 | 43 | 3.09 (2.43–3.94) | 2.71 (2.11–3.47) | 3.28 (1.98–5.49) | 2.04 (1.18–3.53) |
| *P*-value (linear trend) | | | | <0.001 | <0.001 | <0.001 | <0.001 |

[a] Logistic regression estimates with terms for age, sex and centre. [b] Logistic regression estimates with terms for sex, age, centres, hair colour and eye colour.

**Table V** Odds ratios (ORs) of BCC and SCC by number of sunburns and age at first sunburn

| | *No. of controls* | *No. of BCCs* | *No. of SCCs* | *BCC OR[a] (95% CI)* | *BCC OR[b] (95% CI)* | *SCC OR[a] (95% CI)* | *SCC OR[b] (95% CI)* |
|---|---|---|---|---|---|---|---|
| Number of sunburns in a lifetime | | | | | | | |
| Never | 1345 | 1053 | 169 | 1 (Reference) | 1 (Reference) | 1 (Reference) | 1 (Reference) |
| 1 | 305 | 316 | 38 | 1.31 (1.09–1.57) | 1.13 (0.94–1.36) | 1.08 (0.70–1.65) | 0.78 (0.49–1.23) |
| 2 | 65 | 78 | 10 | 1.53 (1.08–2.15) | 1.30 (0.92–1.84) | 1.33 (0.61–2.87) | 0.89 (0.38–2.08) |
| 3+ | 80 | 102 | 11 | 1.65 (1.21–2.24) | 1.30 (0.95–1.78) | 0.86 (0.40–1.85) | 0.54 (0.24–1.21) |
| P-value (linear trend) | | | | <0.001 | 0.031 | 0.298 | 0.529 |
| Age at first sunburn | | | | | | | |
| More than 15 years old or never | 1741 | 1458 | 218 | 1 (Reference) | 1 (Refrence) | 1 (Reference) | 1 (reference) |
| 15 years old or less | 54 | 91 | 10 | 2.05 (1.45–2.88) | 1.68 (1.17–2.39) | 1.90 (0.90–4.03) | 1.26 (0.56–2.80) |

[a]Logistic regression estimates with terms for age, sex and centre. [b] Logistic regression estimates with terms for sex, age, centre, hair colour, eye colour, and skin reaction to sun exposure.

fold to 10-fold increased risk for BCC if they experienced sunburns before age 15 (Table VI). The risk for SCC was substantial in people with fair hair and blue, hazel or grey eyes and a tendency to sunburn, as shown by an odds ratio of 54 for subjects with blue eyes, red hair, who never tanned and always burnt (Table VII).

The final model for pigmentary traits and skin characteristics included terms for hair colour, eye colour and skin reaction to sun exposure in both types of skin cancer, with the highest risks in SCC, whereas young age at first sunburn was present only in the BCC model. Table VIII shows parameter estimates from a logistic model. This model would

**Table VI** Odds ratios (ORs) and 95% confidence intervals (CI) of basal cell carcinoma[a] by age at first sunburn and skin reaction to sun exposure in some high-risk pigmentary traits

| *Hair colour* | | *Black/dark brown* | | *Blonde* | | *Light blonde* | | *Red* | |
|---|---|---|---|---|---|---|---|---|---|
| *Age at first sunburn* | *Skin reaction* | *Dark eyes* | *Blue eyes* | *Dark eyes* | *Blue eyes* | *Dark eyes* | *Blue eyes* | *Dark eyes* | *Blue eyes* |
| More than 15 years old | Tan, no burn | 1 (Reference) | 1.36 (1.15–1.62) | 1.44 (1.07–1.95) | 1.97 (1.45–2.68) | 1.51 (1.08–2.12) | 2.06 (1.47–2.89) | 1.58 (1.09–2.30) | 2.16 (1.49–3.13) |
| 15 years old or less | Sunburn then tan | 2.77 (1.92–3.99) | 3.78 (2.54–5.62) | 4.01 (2.54–6.31) | 5.47 (3.48–8.61) | 4.19 (2.60–6.77) | 5.73 (3.57–9.19) | 4.39 (2.66–7.27) | 6.00 (3.65–9.84) |
| 15 years old or less | Sunburn, never tan | 4.44 (2.94–6.70) | 6.06 (3.92–9.37) | 6.42 (3.97–10.39) | 8.76 (5.44–14.11) | 6.72 (4.07–11.10) | 9.17 (5.60–15.04) | 7.04 (4.17–11.89) | 9.61 (5.74–16.07) |

[a] Logistic regression estimates with terms for sex, age, centre, hair colour, eye colour, skin reaction to sun exposure, and age at first sunburn.

**Table VII** Odds ratios (ORs) and 95% confidence intervals (CI) of squamous cell carcinoma[a] by skin reaction to sun exposure in some high-risk pigmentary traits

| *Hair colour* | *Black/dark brown* | | *Blonde* | | *Light blonde* | | *Red* | |
|---|---|---|---|---|---|---|---|---|
| *Skin reaction* | *Dark eyes* | *Blue eyes* | *Dark eyes* | *Blue eyes* | *Dark eyes* | *Blue eyes* | *Dark eyes* | *Blue eyes* |
| Tan, no burn | 1 (Reference) | 1.78 (1.16–2.74) | 4.34 (1.03–18.25) | 7.75 (1.77–33.88) | 6.79 (1.61–28.61) | 12.11 (2.76–53.19) | 13.97 (2.93–66.75) | 24.93 (4.98–124.72) |
| Sunburn then tan | 1.47 (1.16–1.87) | 2.62 (1.62–4.25) | 6.40 (1.53–26.77) | 11.41 (2.63–49.52) | 9.99 (2.38–41.80) | 17.82 (4.10–77.45) | 20.56 (4.36–97.10) | 36.68 (7.44–180.89) |
| Sunburn, never tan | 2.16 (1.34–3.50) | 3.86 (2.57–7.81) | 9.42 (2.17–40.86) | 16.79 (3.75–75.25) | 14.70 (3.40–63.55) | 26.22 (5.86–117.24) | 30.27 (6.25–146.56) | 53.98 (10.72–271.86) |

[a] Logistic regression estimates with terms for sex, age, centre, hair colour, eye colour, and skin reaction to sun exposure.

**Table VIII** Odds ratios (ORs) of BCC and SCC including independently significant variables and adjusting for age, sex and centre

| | *No. of controls* | *No. of BCCs* | *No. of SCCs* | *BCC OR (95% CI)* | *SCC OR (95% CI)* |
|---|---|---|---|---|---|
| Hair colour | | | | | |
| Black | 154 | 99 | 12 | 1 (Reference) | 1 (Reference) |
| Brown | 699 | 544 | 80 | 1.13 (0.90–1.51) | 1.50 (0.79–2.86) |
| Light/brown | 597 | 514 | 67 | 1.19 (0.89–1.59) | 1.57 (0.81–3.04) |
| Blonde | 253 | 257 | 30 | 1.24 (0.90–1.72) | 1.64 (0.79–3.42) |
| Light blonde | 81 | 121 | 29 | 1.72 (1.16–2.57) | 5.02 (2.30–10.94) |
| Red | 11 | 16 | 10 | 1.31 (0.57–3.03) | 13.00 (4.29–39.38) |
| Eye colour | | | | | |
| Black/dark brown | 542 | 332 | 39 | 1 (Reference) | 1 (Reference) |
| Blue/hazel/grey/green | 1253 | 1217 | 189 | 1.38 (1.16–1.63) | 1.65 (1.11–2.43) |
| Skin reaction to sun exposure | | | | | |
| Tan, no burn | 518 | 299 | 44 | 1 (Reference) | 1 (Reference) |
| Burn, then tan | 1088 | 943 | 71 | 1.49 (1.26–1.78) | 1.35 (0.93–1.96) |
| Burn, never tan | 184 | 299 | 43 | 2.70 (2.10–3.47) | 1.97 (1.19–3.26) |
| Age at first sunburn | | | | | |
| More than 15 years old or never | 1741 | 1458 | 218 | 1 (Reference) | – |
| 15 years old or less | 54 | 91 | 10 | 1.65 (1.16–2.36) | – |

also be useful in building a parsimonious set of controlling variables that can make up the basis for testing other risk factors.

### Discussion

Previous results have shown the relationship between sun exposure, skin characteristics and non-melanocytic skin cancer, but they were mainly based on Anglo-Saxon populations. We investigated several risk factors in a wide south European population in which different skin types, sun exposure patterns and histological subtypes confirmed by a panel of pathologists are sufficiently represented for statistical purposes.

The anatomical site distribution, with a substantial proportion of BCC on the trunk (14.1% in men and 10.1% in women), is consistent with previous observations in the Canton of Vaud, Switzerland (Levi *et al.*, 1988), The Netherlands (Coebergh *et al.*, 1991) and Tasmania (Kaldor *et al.*, 1993), although to a lesser extent in Norway (27.6% in men and 25.2% in women) (Magnus, 1991) and in Western Australia (32% in men and 21% in women) (Kricker *et al.*, 1990). Indeed, data from Western Australia were collected through a specific survey, whereas other studies relied upon routinely collected data from cancer registries. Although the anatomical site distribution of SCC was rather stable over time, several surveys showed that BCC lesions on the trunk increased in the last decade (Fears and Scotto, 1982; Levi *et al.*, 1988; Gallagher *et al.*, 1990; Coebergh *et al.*, 1991; Magnus, 1991).

Skin characteristics are considered as risk indicators for skin cancer as sun exposure produces skin cancer at different rates for skin types with different sun sensitivity. In general, apart from rare forms of skin cancer (basal cell syndrome in xeroderma pigmentosum), sun exposure is considered essential in inducing skin cancers. Nevertheless, identification of high-risk groups through easily detectable skin characteristics can help in targeting preventive interventions.

In the present study, we used hair colour, eye colour and skin reaction to sun exposure as indicators of skin type. These indicators have the advantage of being easier to use and more accurate than skin colour, and are therefore, more suitable for risk assessment and in messages to the public. Conversely, skin colour is difficult to measure reliably either subjectively or objectively. Although a high correlation would be expected between pigmentary traits, our results showed an independent effect, even if estimates were sensibly attentuated after adjustment for mutual confounding effects.

Comparison with other studies is made difficult by phenotype differences between study populations and the use of different measurement scales. Indeed, given the characteristics of the Mediterranean population, it is possible that in the baseline category our study included subjects with darker complexion than other studies. In general, eye colour and skin sun sensitivity showed similar results in both BCC and SCC with OR for eye colour ranging from 1.2 (Hogan *et al.*, 1989; Kricker *et al.*, 1991) to 3.4 (Vitasa *et al.*, 1990), and ORs for sun sensitivity ranging from 1.3 in subjects with 'an average tan' (Hunter *et al.*, 1990) to 6.1 in subjects who 'always burn' (Marks *et al.*, 1993).

For hair colour other studies used less expanded scales with similar results for BCC (Hogan *et al.*, 1989; Hunter *et al.*, 1990; Green and Battistutta, 1990; Kricker *et al.*, 1991). Odds ratios for SCC lower than ours, particularly in subjects with red hair, were however found with a maximum OR of 3.3 in a study based on only 21 SCCs (Green and Battistutta, 1990).

The lower ORs found for eye colour, as compared with hair colour, might simply indicate the greater difficulty in assessing eye colour with consequently larger measurement error.

Although, number of sunburns life-long is very difficult to recall and can be seriously underestimated, history of sunburns can be considered a comprehensive indicator of skin sensitivity and sun exposure, as sunburn is caused by an exposure that exceeds the skin's reparation ability. Few sunburns life-long, therefore, can indicate true skin resistance as well as a tendency to avoid sun exposure as a result of skin sensitivity. In our study the relationship with number of sunburns life-long was present only in BCC with OR similar to those found elsewhere (Hogan *et al.*, 1989; Hunter *et al.*, 1990; Kricker *et al.*, 1995*a*). However, the association was not significant after controlling for skin characteristics. Allowance for these variables is open to criticism as sunburns, as previously noted, occur only if exposure exceeds skin protection (Green *et al.*, 1985; Kricker *et al.*, 1995*a*).

Age at sunburn has been replaced, as a proxy, by age of arrival in sunny areas. For example, in previous studies in Australia it was found that immigration at age 10 years or less implied a slight risk increase (Armstrong, 1983; Kricker *et al.*, 1991). A recent study reported a similar risk increase for severe sunburns in childhood (Gallagher *et al.*, 1995*a*), and another study estimated that the strongest effect of sunburns on BCC was at 10–14 years of age (Kricker *et al.*, 1995*a*). Again, young age at sunburn is an indicator not only of the direct effect of sun exposure, but also of early starting of heavy exposure or a sign of sun sensitivity in subjects who can develop a darker and less sensitive complexion with ageing. This variable, therefore, can explain the marginal contribution to the risk of BCC in subjects not belonging to traditional high-risk groups. This result also mimics what has been found in cutaneous melanoma, in which history of past sunburn during childhood has been associated with a 2-fold increased risk (Østerlind, *et al.*, 1988; Elwood *et al.*, 1990; Zanetti *et al.*, 1992; Weinstock *et al.*, 1991).

Although SCC occurs mainly in old age, we restricted eligibility to 20–70 years because of the difficulty in gathering reliable information in elderly subjects; nevertheless, we were able to collect 228 SCC cases. The discrepancy between median incidence age of SCC in the general population and the median age in our data set is unlikely to bias the present results, as the control sample was balanced for age with cases and the residual effect of age was allowed for by adjusting odds ratios for the exact annual age.

Another possible source of bias was the different population bases of the control sample; although a certain degree of distortion cannot be completely ruled out, we checked for consistency among centres, and by aggregating centres by country. Country proved to be a stronger confounder than study design (hospital or population basis).

Compliance among cases was high for all centres. We had a lower response rate among controls in Besançon (55.6%) and Turin (63.6%) although still similar to those in other population-based case–control studies (Engel *et al.*, 1988; Green *et al.*, 1988; Hogan *et al.*, 1989; Marks *et al.*, 1989; Hunter *et al.*, 1990; Vitasa *et al.*, 1990). However, the interview setting was similar for cases and controls (at home or outpatient clinics), thus minimising possible sources of bias.

In summary, the present study confirms the role of constitutional factors in high-risk groups for both BCC and SCC. However, a somewhat stronger association with phenotypic characteristics emerged for SCC than for BCC. Conversely, sunburns were a more important risk factor for BCC than for SCC, partly as a consequence of the different pattern of sun exposure relevant to these two skin cancer types, which will be discussed in a companion paper (Rosso *et al.*, 1996).

**Acknowledgements**

This study has been supported by a research grant from Europe Against Cancer (contract nos. 890139, 910539, 920584) and by Associazione Italiana per la Ricerca sul Cancro (AIRC), Italy; Fondo de Investigacion Sanitaria de la Securidad Social (FISS), Spain (contract no. 90E0720); Ligue Nationale Contre le Cancer, France; Consiglio Nazionale delle Ricerche (CNR), Italy; Institut National de la Santé et de la Recerche Médicale (INSERM), France. We thank Mrs M Casale for her valuable help in preparing the questionnaire and in training interviewers and Dr BK Armstrong for his useful comments on the manuscripts. We are also grateful to the numerous colleagues, dermatologists, pathologists and general practitioners who allowed access to patients and histological material.

### References

ARMSTRONG BK, WOODINGS T, STENHOUSE NS AND McCALL MG. (1983). *Mortality from Cancer in Migrants to Australia 1962–1971*. University of Western Australia: Perth.

COEBERGH JWW, NEUMANN HAM, VRINTS LW, VAN DER HEIJDEN L, MEIJER WJ AND VERHAGEN TEULINGS MT. (1991). Trends in the incidence of non-melanoma skin cancer in the SE Netherlands 1975–1988: a registry-based study. *Br. J. Dermatol.*, **125**, 353–359.

ELWOOD JM, WHITHEAD SM, DAVISON J, STEWART M AND GALT M. (1990). Malignant melanoma in England: risks associated with naevi, freckles, social class, hair colour and sunburn. *Int. J. Epidemiol.*, **19**, 801–810.

ENGEL A, JOHNSON ML AND HAYNES SG. (1988). Health effects of sunlight exposure in the United States. *Arch. Dermatol.*, **124**, 72–79.

FEARS TR AND SCOTTO J. (1982). Changes in skin cancer morbidity between 1971–72 and 1977–78. *J. Natl Cancer Inst.*, **69**, 365–370.

GALLAGHER RP, MA B, McLEAN DI, YANG CP, HO V, CARRUTHERS JA AND WARSHAWSKI LM. (1990). Trends in basal cell carcinoma, squamous cell carcinoma, and melanoma of the skin from 1973 through 1987. *J. Am. Acad. Dermatol.*, **23**, 413–421.

GALLAGHER RP, HILL GB, BAJDIK CD, FINCHAM S, COLDMAN AJ, McLEAN DI AND THRELFALL WJ. (1995*a*). Sunlight exposure, pigmentary factors, and risk of nonmelanocytic skin cancer. I. Basal cell carcinoma. *Arch. Dermatol.*, **131**, 157–163.

GALLAGHER RP, HILL GB, BAJDIK CD, FINCHAM S, COLDMAN AJ, McLEAN DI AND THRELFALL WJ. (1995*b*). Sunlight exposure, pigmentary factors, and risk of nonmelanocytic skin cancer. II. Squamous cell carcinoma. *Arch. Dermatol.*, **131**, 164–169.

GREEN AC AND BATTISTUTTA D. (1990). Incidence and determinants of skin cancer in a high-risk Australian population. *Int. J. Cancer*, **46**, 356–361.

GREEN AC AND MARTIN NG. (1990). Measurement and perception of skin colour in a skin cancer survey. *Br. J. Dermatol.*, **123**, 77–84.

GREEN AC, SISKIND V AND BAIN C. (1985). Sunburns and malignant melanoma. *Br. J. Cancer*, **51**, 393–397.

GREEN AC, BEARDMORE G, HART V, LESLIE D AND MARKS R. (1988). Skin cancer in a Queensland population. *J. Am. Acad. Dermatol.*, **19**, 1045–1052.

HOGAN DJ, TO T, GRAN L, WONG D AND LANE PR. (1989). Risk factors for basal cell carcinoma. *Int. J. Dermatol.*, **28**, 591–594.

HUNTER DJ, COLDITZ GA, STAMPFER MJ, ROSNER B, WILLET WC AND SPEIZER FE. (1990). Risk factors for basal cell carcinoma in a prospective cohort of women. *Ann. Epidemiol.*, **1**, 13–23.

INTERNATIONAL AGENCY FOR RESEARCH ON CANCER. (1992). *IARC Monographs on the Evaluation of Carcinogenic risks to Humans, Vol. 55, Solar and Ultraviolet Radiation*. IARC: Lyon.

KALDOR J, SHUGG D, YOUNG B, DWYER T AND WANG YG. (1993). Non-melanoma skin cancer: ten years of cancer-registry-based surveillance. *Int. J. Cancer*, **53**, 886–891.

KRICKER A, ENGLISH DR, RANDELL PL, HEENAN PJ, CLAY CD, DELANEY TA AND ARMSTRONG BK. (1990). Skin cancer in Geraldton, Western Australia: a survey of incidence and prevalence. *Med. J. Aust.*, **152**, 399–407.

KRICKER A, ARMSTRONG BK, ENGLISH DR AND HEENAN PJ. (1991). Pigmentary and cutaneous risk factors for non-melanocytic skin cancer–a case–control study. *Int. J. Cancer*, **48**, 650–662.

KRICKER A, ARMSTRONG BK AND ENGLISH DR. (1994). Sun exposure and non-melanocytic skin cancer. *Cancer Causes Control*, **5**, 367–392.

KRICKER A, ARMSTRONG BK, ENGLISH DR AND HEENAN PJ. (1995*a*). Does intermittent sun exposure cause basal cell carcinoma? A case–control study in Western Australia. *Int. J. Cancer*, **60**, 489–494.

KRICKER A, ARMSTRONG BK, ENGLISH DR AND HEENAN PJ. (1995*b*). A dose–response curve for sun exposure and basal cell carcinoma. *Int. J. Cancer*, **60**, 482–488.

KUBASIEWICZ M, STARZYNSKI Z AND SZYMCZACK W. (1991). Case–referent study on skin cancer and its relation to occupational exposure to polycyclic aromatic hydrocarbons. II–Study results. *Pol. J. Occup. Med.*, **4**, 141–147.

LEVI F, LA VECCHIA C, TE V AND MEZZANOTTE G. (1988). Descriptive epidemiology of skin cancer in the Swiss Canton of Vaud. *Int. J. Cancer*, **42**, 811–816.

MAGNUS K. (1991). The Nordic profile of skin cancer incidence. A comparative epidemiological study of the three main types of skin cancer. *Int. J. Cancer*, **47**, 12–19.

MARKS R, JOLLEY D, DOREVITCH AP AND SELWOOD TS. (1989). The incidence of non-melanocytic skin cancers in an Australian population: results of a five-year prospective study. *Med. J. Aust.*, **150**, 475–478.

MARKS R, STAPLES M AND GILES GG. (1993). Trends in non-melanocytic skin cancer treated in Australia: the second national survey. *Int. J. Cancer*, **53**, 585–590.

OETTLÉ AG. (1963). Skin cancer in Africa. *Natl Cancer Inst. Monogr.*, **10**, 197–214.

ØSTERLIND A, TUCKER MA, STONE BJ AND JENSEN OM. (1988). The Danish case–control study of cutaneous malignant melanoma. II. Importance of UV-light exposure. *Int. J. Cancer*, **42**, 319–324.

PARKIN DM, MUIR CS, WHELAN SL, GAO Y-T, FERLAY J AND POWELL J. (1992). *Cancer Incidence in Five Continents*, Vol. VI. IARC Scientific Publications No. 120. IARC: Lyon.

PREGIBON D. (1981). Logistic regression diagnostics. *Ann. Stat.*, **9**, 705–724.

RON E, MODAN B, PRESTON D, ALFANDARY E, STOVALL M AND BOICE JR JD. (1991). Radiation-induced skin carcinomas of the head and neck. *Radiat. Res.*, **125**, 318–325.

ROSSO S, ZANETTI R, MARTINEZ C, TORMO MJ, SCHRAUB S, SANCHO-GARNIER H, FRANCESCHI S, GAFÀ L, PEREA E, NAVARRO C, LAURENT R, SCHRAMECK C, TALAMINI M, TUMINO R AND WECHSLER J. (1996). The multicentre south European study 'Helios' II: different sun exposure patterns in the aetiology of basal cell and squamous cell carcinomas of the skin. *Br. J. Cancer*, **73**, 1447–1454.

STENBECK KD, BALANDA KP, WILLIAMS MJ AND RING IT. (1990). Patterns of treated non-melanoma skin cancer in Queensland–the region with the highest incidence rates in the world. *Med. J. Aust.*, **153**, 511–515.

VITASA BC, TAYLOR HR, STRICKLAND PT, ROSENTHAL FS, WEST S, ABBEY H, NG SK, MUNOZ B AND EMMET EA. (1990). Association of non-melanoma skin cancer and actinic keratosis with cumulative solar ultraviolet exposure in Maryland watermen. *Cancer*, **65**, 2811–2817.

WEINSTOCK MA, COLDITZ GA, WILLET WC, STAMPFER MJ, BRONSTEIN BR, MIHM MC AND SPEIZ FE. (1991). Melanoma and sun: the effect of swimsuit and a 'Healthy' tan on the risk of non-familial malignant melanoma in women. *Am. J. Epidemiol.*, **134**, 462–470.

ZANETTI R, FRANCESCHI S, ROSSO S, COLONNA S, BIDOLI E. (1992). Cutaneous melanoma and sunburns in childhood in a southern European population. *Eur. J. Cancer*, **28A**, 1172–1176.


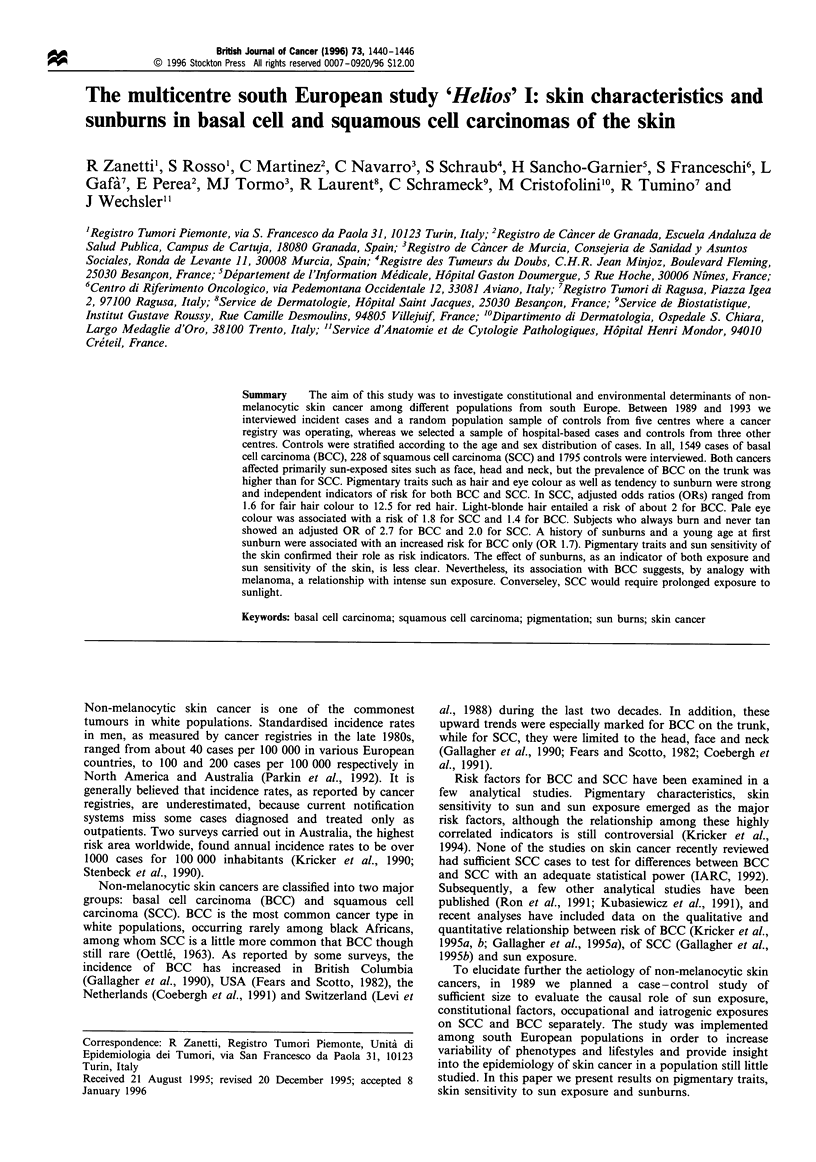

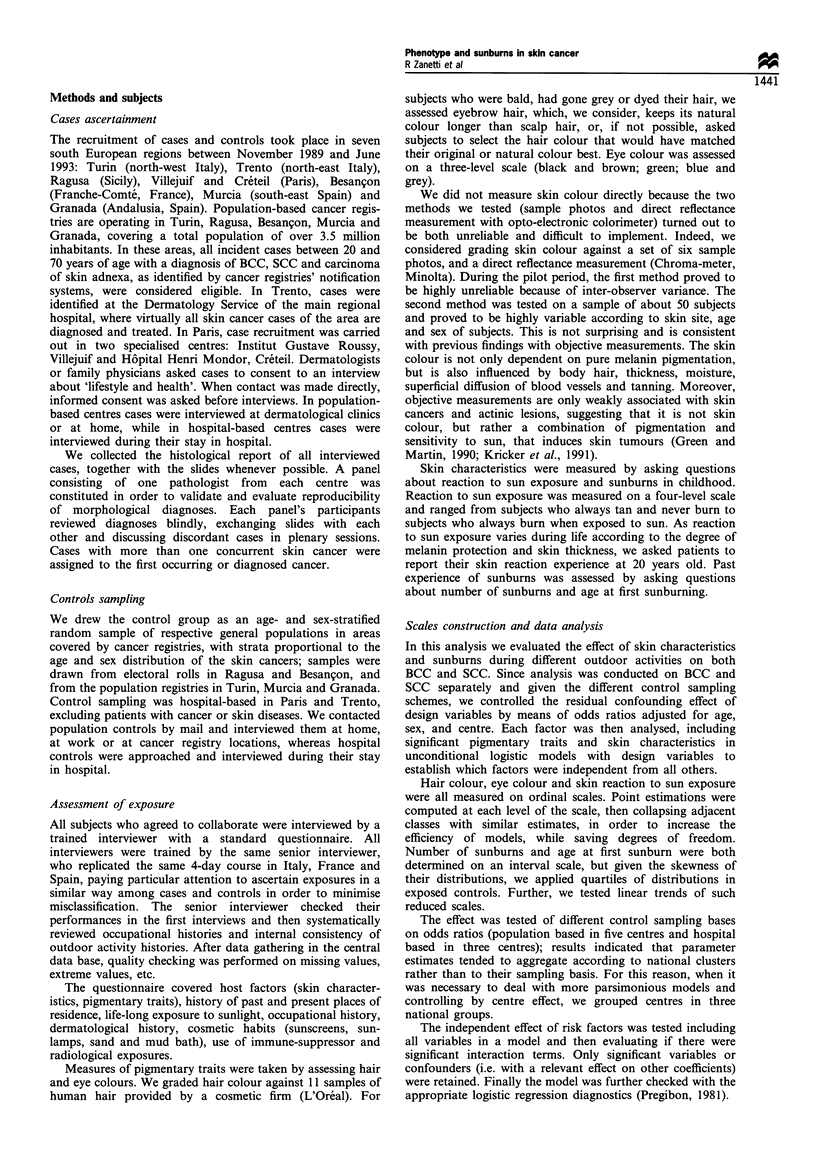

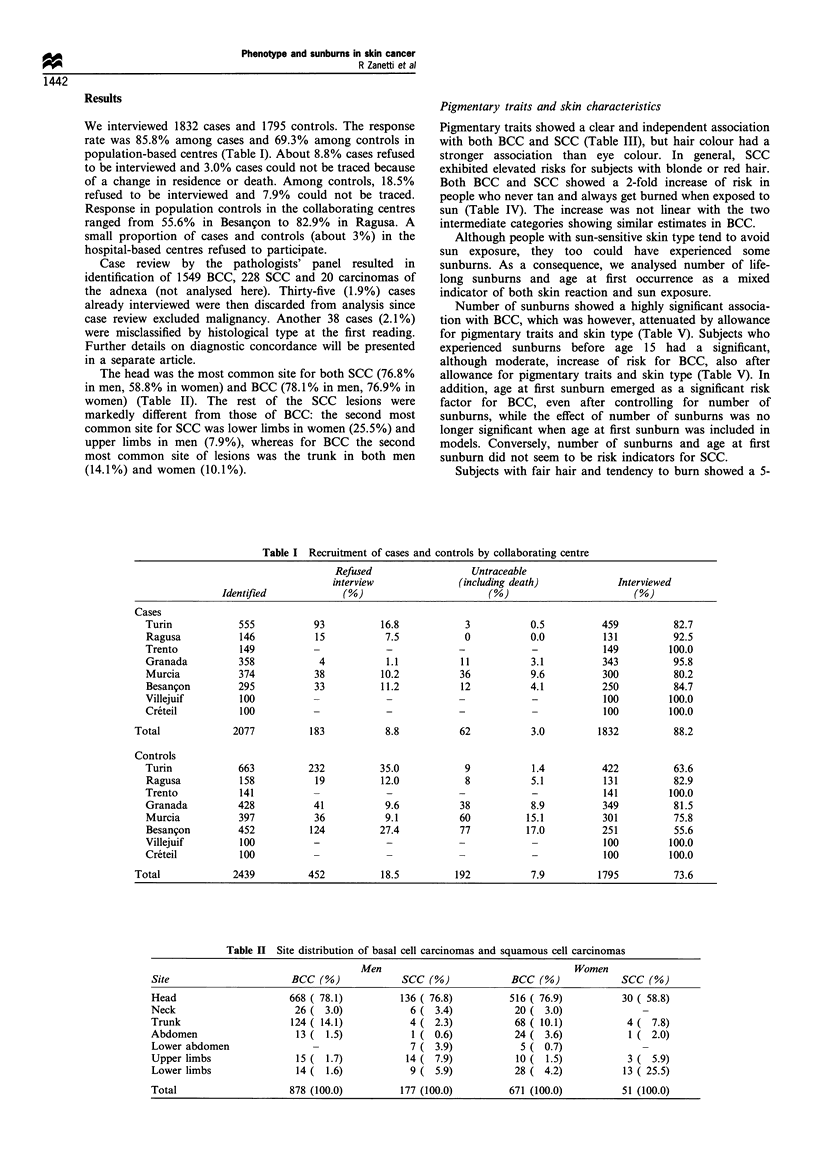

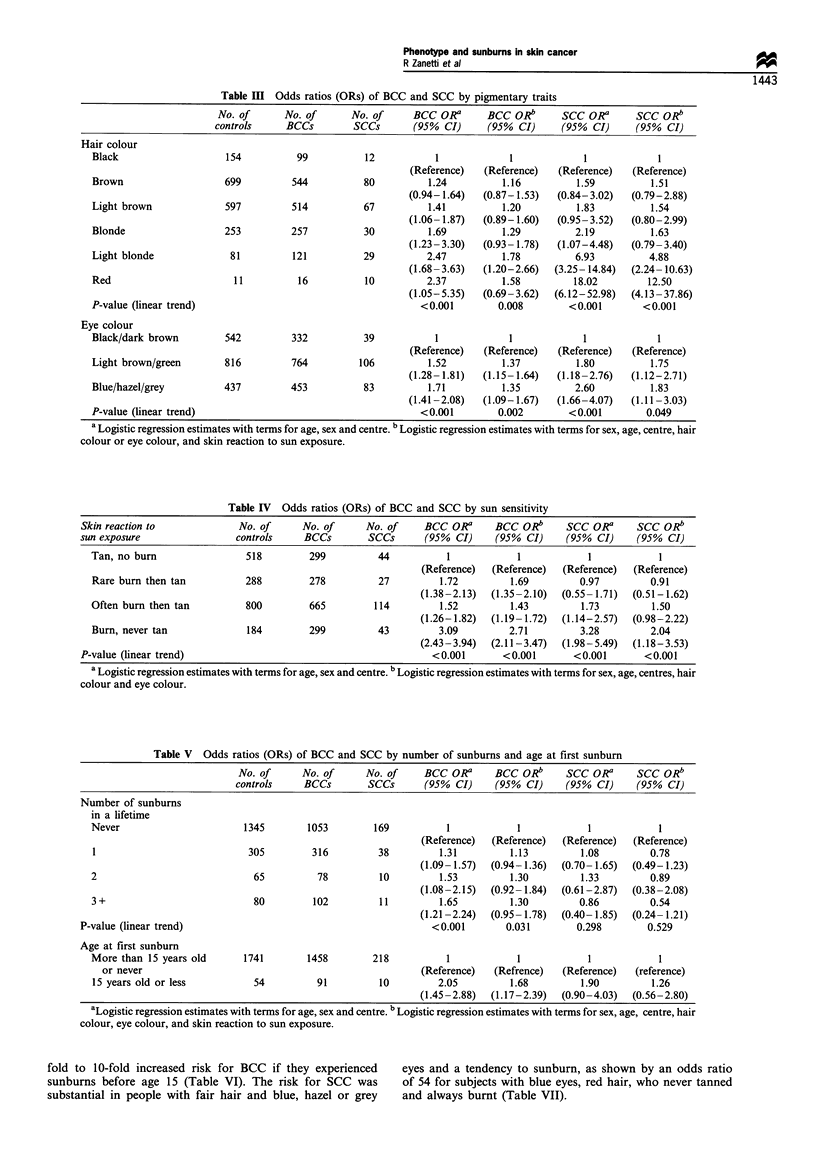

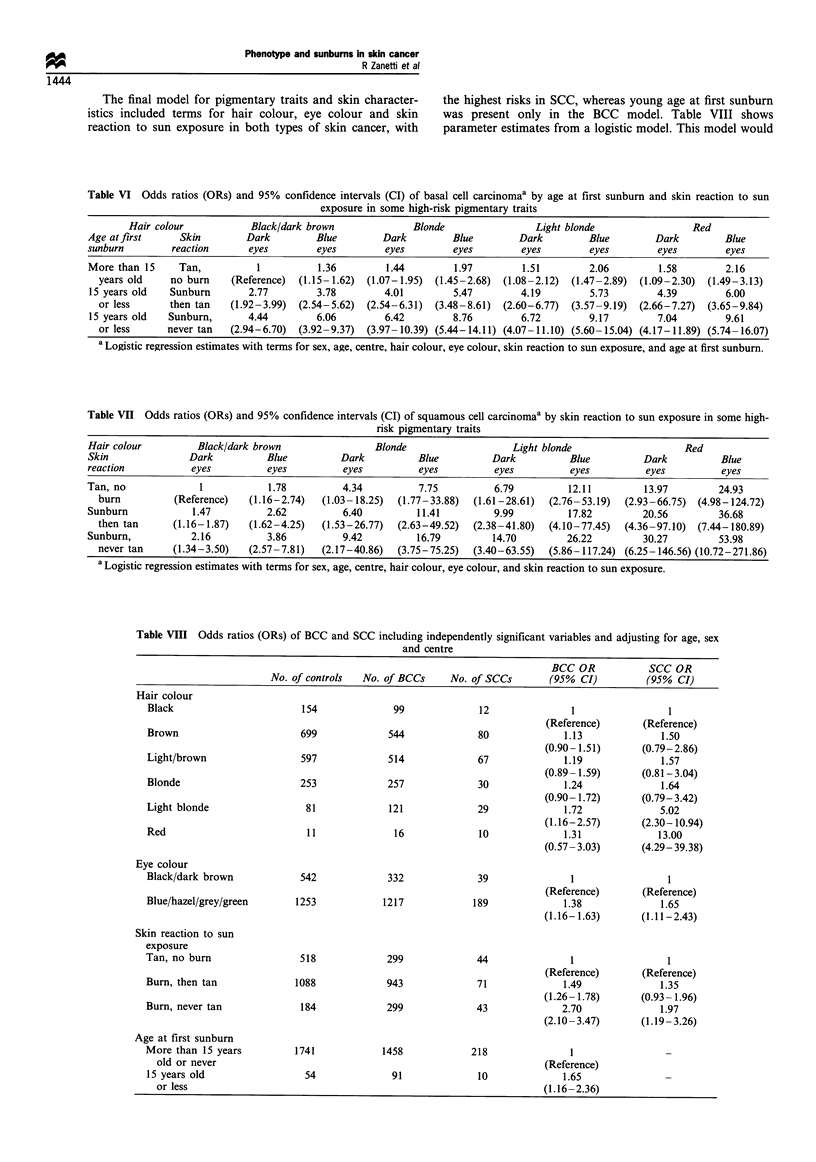

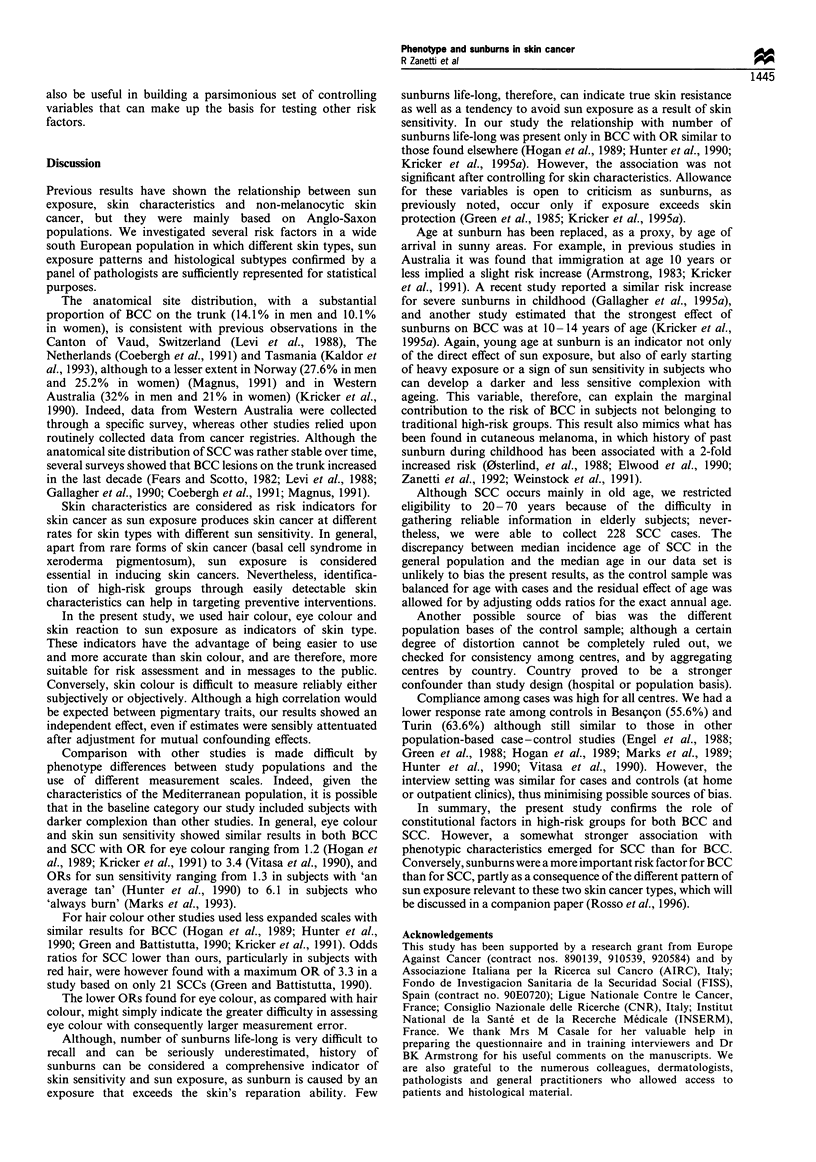

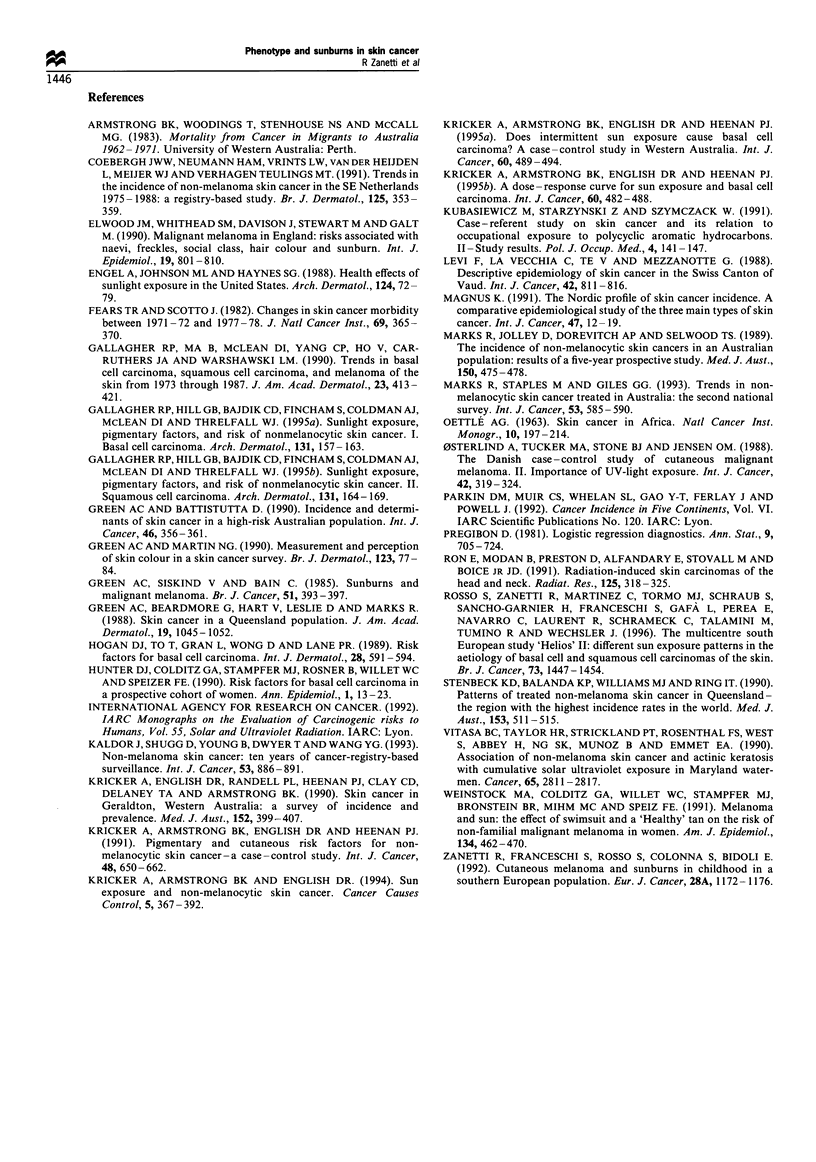


## References

[OCR_00865] Andersen B. R., Van Epps D. E. (1972). Suppression of chemotatic activity of human neutrophils by streptolysin O.. J Infect Dis.

[OCR_00863] Coebergh J. W., Neumann H. A., Vrints L. W., van der Heijden L., Meijer W. J., Verhagen-Teulings M. T. (1991). Trends in the incidence of non-melanoma skin cancer in the SE Netherlands 1975-1988: a registry-based study.. Br J Dermatol.

[OCR_00868] Elwood J. M., Whitehead S. M., Davison J., Stewart M., Galt M. (1990). Malignant melanoma in England: risks associated with naevi, freckles, social class, hair colour, and sunburn.. Int J Epidemiol.

[OCR_00876] Engel A., Johnson M. L., Haynes S. G. (1988). Health effects of sunlight exposure in the United States. Results from the first National Health and Nutrition Examination Survey, 1971-1974.. Arch Dermatol.

[OCR_00881] Fears T. R., Scotto J. (1982). Changes in skin cancer morbidity between 1971-72 and 1977-78.. J Natl Cancer Inst.

[OCR_00897] Gallagher R. P., Hill G. B., Bajdik C. D., Coldman A. J., Fincham S., McLean D. I., Threlfall W. J. (1995). Sunlight exposure, pigmentation factors, and risk of nonmelanocytic skin cancer. II. Squamous cell carcinoma.. Arch Dermatol.

[OCR_00894] Gallagher R. P., Hill G. B., Bajdik C. D., Fincham S., Coldman A. J., McLean D. I., Threlfall W. J. (1995). Sunlight exposure, pigmentary factors, and risk of nonmelanocytic skin cancer. I. Basal cell carcinoma.. Arch Dermatol.

[OCR_00887] Gallagher R. P., Ma B., McLean D. I., Yang C. P., Ho V., Carruthers J. A., Warshawski L. M. (1990). Trends in basal cell carcinoma, squamous cell carcinoma, and melanoma of the skin from 1973 through 1987.. J Am Acad Dermatol.

[OCR_00903] Green A., Battistutta D. (1990). Incidence and determinants of skin cancer in a high-risk Australian population.. Int J Cancer.

[OCR_00917] Green A., Beardmore G., Hart V., Leslie D., Marks R., Staines D. (1988). Skin cancer in a Queensland population.. J Am Acad Dermatol.

[OCR_00908] Green A., Martin N. G. (1990). Measurement and perception of skin colour in a skin cancer survey.. Br J Dermatol.

[OCR_00915] Green A., Siskind V., Bain C., Alexander J. (1985). Sunburn and malignant melanoma.. Br J Cancer.

[OCR_00922] Hogan D. J., To T., Gran L., Wong D., Lane P. R. (1989). Risk factors for basal cell carcinoma.. Int J Dermatol.

[OCR_00929] Hunter D. J., Colditz G. A., Stampfer M. J., Rosner B., Willett W. C., Speizer F. E. (1990). Risk factors for basal cell carcinoma in a prospective cohort of women.. Ann Epidemiol.

[OCR_00938] Kaldor J., Shugg D., Young B., Dwyer T., Wang Y. G. (1993). Non-melanoma skin cancer: ten years of cancer-registry-based surveillance.. Int J Cancer.

[OCR_00964] Kricker A., Armstrong B. K., English D. R., Heenan P. J. (1995). A dose-response curve for sun exposure and basal cell carcinoma.. Int J Cancer.

[OCR_00960] Kricker A., Armstrong B. K., English D. R., Heenan P. J. (1995). Does intermittent sun exposure cause basal cell carcinoma? a case-control study in Western Australia.. Int J Cancer.

[OCR_00949] Kricker A., Armstrong B. K., English D. R., Heenan P. J. (1991). Pigmentary and cutaneous risk factors for non-melanocytic skin cancer--a case-control study.. Int J Cancer.

[OCR_00953] Kricker A., Armstrong B. K., English D. R. (1994). Sun exposure and non-melanocytic skin cancer.. Cancer Causes Control.

[OCR_00944] Kricker A., English D. R., Randell P. L., Heenan P. J., Clay C. D., Delaney T. A., Armstrong B. K. (1990). Skin cancer in Geraldton, Western Australia: a survey of incidence and prevalence.. Med J Aust.

[OCR_00969] Kubasiewicz M., Starzyński Z., Szymczak W. (1991). Case-referent study on skin cancer and its relation to occupational exposure to polycyclic aromatic hydrocarbons. II. Study results.. Pol J Occup Med Environ Health.

[OCR_00975] Levi F., La Vecchia C., Te V. C., Mezzanotte G. (1988). Descriptive epidemiology of skin cancer in the Swiss Canton of Vaud.. Int J Cancer.

[OCR_00987] Marks R., Jolley D., Dorevitch A. P., Selwood T. S. (1989). The incidence of non-melanocytic skin cancers in an Australian population: results of a five-year prospective study.. Med J Aust.

[OCR_00993] Marks R., Staples M., Giles G. G. (1993). Trends in non-melanocytic skin cancer treated in Australia: the second national survey.. Int J Cancer.

[OCR_01002] Osterlind A., Tucker M. A., Stone B. J., Jensen O. M. (1988). The Danish case-control study of cutaneous malignant melanoma. II. Importance of UV-light exposure.. Int J Cancer.

[OCR_01015] Ron E., Modan B., Preston D., Alfandary E., Stovall M., Boice J. D. (1991). Radiation-induced skin carcinomas of the head and neck.. Radiat Res.

[OCR_01024] Rosso S., Zanetti R., Martinez C., Tormo M. J., Schraub S., Sancho-Garnier H., Franceschi S., Gafà L., Perea E., Navarro C. (1996). The multicentre south European study 'Helios'. II: Different sun exposure patterns in the aetiology of basal cell and squamous cell carcinomas of the skin.. Br J Cancer.

[OCR_01031] Stenbeck K. D., Balanda K. P., Williams M. J., Ring I. T., MacLennan R., Chick J. E., Morton A. P. (1990). Patterns of treated non-melanoma skin cancer in Queensland--the region with the highest incidence rates in the world.. Med J Aust.

[OCR_01035] Vitasa B. C., Taylor H. R., Strickland P. T., Rosenthal F. S., West S., Abbey H., Ng S. K., Munoz B., Emmett E. A. (1990). Association of nonmelanoma skin cancer and actinic keratosis with cumulative solar ultraviolet exposure in Maryland watermen.. Cancer.

[OCR_01042] Weinstock M. A., Colditz G. A., Willett W. C., Stampfer M. J., Bronstein B. R., Mihm M. C., Speizer F. E. (1991). Melanoma and the sun: the effect of swimsuits and a "healthy" tan on the risk of nonfamilial malignant melanoma in women.. Am J Epidemiol.

[OCR_01049] Zanetti R., Franceschi S., Rosso S., Colonna S., Bidoli E. (1992). Cutaneous melanoma and sunburns in childhood in a southern European population.. Eur J Cancer.

